# Costs of sleep apnoea treatment can be reduced

**DOI:** 10.7196/AJTCCM.2021.v27i3.163

**Published:** 2021-10-04

**Authors:** R Farré, D Gozal, J M Montserrat

**Affiliations:** 1 Unitat de Biofísica i Bioenginyeria, Facultat de Medicina i Ciències de la Salut, Universitat de Barcelona, Barcelona, Spain; 2 CIBER de Enfermedades Respiratorias, Madrid, Spain; 3 Institut Investigacions Biomediques August Pi Sunyer, Barcelona, Spain; 4 Department of Child Health, The University of Missouri School of Medicine, Columbia, Missouri, USA; 5 Sleep Lab, Hospital Clinic, Universitat de Barcelona, Barcelona, Spain

## Editorial

**Dear Editor,**

**Fig. 1:**
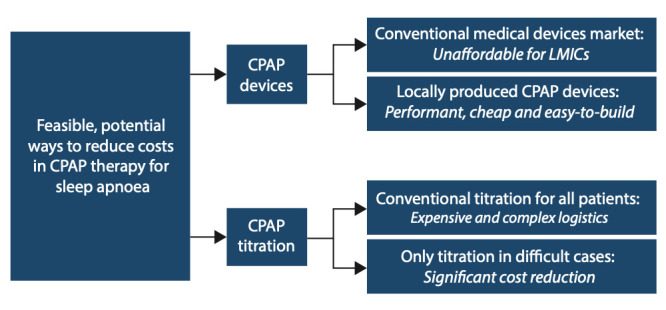
Diagram showing feasible, potential ways to reduce costs in CPAP therapy for sleep apnoea CPAP = continuous positive airway pressure LMICs = low- and middle-income countries

A recent editorial in the *AJTCCM*^[1]^ asked whether it was affordable to treat sleep apnoea in most African clinical settings. After mentioning the epidemiological impact of sleep apnoea in African countries, the author focused on the high cost of treatment devices, particularly those providing continuous positive airway pressure (CPAP), the most widely used and most effective therapeutic modality for sleep apnoea. The fact that CPAP devices should be imported from developed countries makes this therapy simply unaffordable for the public health system and too onerous for most individual patients in low- and middle-income countries (LMICs). It becomes readily apparent that unless CPAP devices are built in a way that is outside the realm of unaffordable conventional medical devices and market costs, the problem will remain unresolved. Fortunately, there is a potential way to sort this problem out, as CPAP devices are extremely simple from a technological viewpoint. Indeed, it is possible to build CPAP devices at very low cost, while using an extremely simple technological platform, both in terms of infrastructure and personnel.^[2,3]^ We have recently shown that highly performant non-invasive bilevel pressure ventilators (which include CPAP options for sleep apnoea) can be simply and cheaply built following the free, yet detailed technical descriptions provided.^[4]^ Whereas cost of CPAP devices is a remarkable problem, there are other issues that contribute to the high cost of CPAP therapy. Conventional procedures for CPAP implementation in patients require a process in which the optimal CPAP pressure needs to be individually titrated, a process that is onerous in terms of both equipment and staff. Whereas, individual titration is certainly required for some more complex patients, it has been recently proposed that conventional titration can be avoided for the majority of patients.^[5]^ Indeed, epidemiological data on the treatment of 16 780 patients showed that after completing the proccess of individualising labour-intensive and expensive CPAP titrations, 86.4% of sleep apnoea patients were ultimately prescribed and treated with CPAP settings within the range of 7 - 11 cmH_2_O. Accordingly, we proposed that in case that personalised CPAP titration may impede or substantially delay treatment, pressure should be initially prescribed at 9 cmH_2_O for all recently diagnosed obstructive sleep apnoea patients eligible for CPAP, and that in the relatively infrequent case when the patient still manifests residual symptoms, then a visit to the healthcare staff would be needed to modify the CPAP settings.^[5]^ We think that focusing on steps consisting of affordable CPAP device availability and streamlined clinical management approaches (Fig. 1) could radically reduce healthcare costs and markedly improve access to diagnostics and therapy for sleep apnoea in African countries.
